# Optical Property Mapping of Apples and the Relationship With Quality Properties

**DOI:** 10.3389/fpls.2022.873065

**Published:** 2022-04-25

**Authors:** Hehuan Peng, Chang Zhang, Zhizhong Sun, Tong Sun, Dong Hu, Zidong Yang, Jinshuang Wang

**Affiliations:** ^1^College of Optical, Mechanical and Electrical Engineering, Zhejiang A&F University, Hangzhou, China; ^2^Office of Educational Administration, Zhejiang A&F University, Hangzhou, China; ^3^College of Mathematics and Computer Science, Zhejiang A&F University, Hangzhou, China; ^4^Key Laboratory of Crop Harvesting Equipment Technology of Zhejiang Province, Jinhua, China

**Keywords:** optical property mapping, spatial-frequency domain imaging, apple, quality, correlation, prediction

## Abstract

This paper reports on the measurement of optical property mapping of apples at the wavelengths of 460, 527, 630, and 710 nm using spatial-frequency domain imaging (SFDI) technique, for assessing the soluble solid content (SSC), firmness, and color parameters. A laboratory-based multispectral SFDI system was developed for acquiring SFDI of 140 “Golden Delicious” apples, from which absorption coefficient (*μ_*a*_*) and reduced scattering coefficient (*μ_*s*_′*) mappings were quantitatively determined using the three-phase demodulation coupled with curve-fitting method. There was no noticeable spatial variation in the optical property mapping based on the resulting effect of different sizes of the region of interest (ROI) on the average optical properties. Support vector machine (SVM), multiple linear regression (MLR), and partial least square (PLS) models were developed based on *μ_*a*_*, *μ_*s*_′* and their combinations (*μ_*a*_* × *μ_*s*_′* and *μ_*eff*_*) for predicting apple qualities, among which SVM outperformed the best. Better prediction results for quality parameters based on the *μ_*a*_* were observed than those based on the *μ_*s*_′*, and the combinations further improved the prediction performance, compared to the individual *μ_*a*_* or *μ_*s*_′*. The best prediction models for SSC and firmness parameters [slope, flesh firmness (FF), and maximum force (Max.F)] were achieved based on the *μ_*a*_* × *μ_*s*_′*, whereas those for color parameters of b* and C* were based on the *μ_*eff*_*, with the correlation coefficients of prediction as 0.66, 0.68, 0.73, 0.79, 0.86, and 0.86, respectively.

## Introduction

The apple, famous for its rich vitamin and mineral with high-nutritional value, is one of the most consumed fruits worldwide. Apples are available throughout the year due to the advanced and strict storage control. After harvesting, apples are transported immediately from the orchard to the shed storage. Alternatively, apples are graded and sorted first in the warehouse, after which they are directly moved to the cold shed storage (Zhang et al., 2020, 2021). The latter case is more popular because it can meet the increasing and diverse demands of consumers for apple quality. Quality evaluation on intact apple fruit, which is the key step in the process of grading and sorting, is gaining tremendous interest and attention in the field of non-destructive inspection. In the past decades, visible and near-infrared (Vis/NIR) spectroscopy has been widely developed and adopted for quality assessment of plant and food products (Zhao et al., 2020; Daniels et al., 2021; Li and Zhang, 2021; van Wyngaard et al., 2021; Wan et al., 2021), thanks to its rapid and non-invasive character. A large number of statistical models based on Vis/NIR spectroscopy have been built for predicting apple quality properties, such as firmness, crispness, soluble solid content (SSC), and acidity (Gao et al., 2016; Fan et al., 2020; Huang et al., 2020; Tian et al., 2020). However, these models are often valid for the conditions under which they were trained and are not applicable under very variable conditions, such as different cultivars, batches, and places of origin. The main reason causing the weak robustness and applicability of the statistical models is that conventional Vis/NIR spectroscopy typically relates the spectra to the chemical and/or physical properties using a “black-box” method directly and it cannot offer separate information on the absorption and scattering properties of plant and food tissues. It is thus desirable to reveal the insight into light-tissue interaction (mainly absorption and multiple scattering), which is expected to provide more reliable prediction of quality properties under very variable conditions.

In the last decade, optical properties of fruits and vegetables, mainly referring to absorption coefficient (*μ_*a*_*, mm^–1^) and reduced scattering coefficient (*μ_*s*_′*, mm^–1^), have been measured by different researchers using diverse optical sensing techniques (Hu et al., 2015; Lu et al., 2020), such as integrating sphere (IS), time-resolved (TR), spatially resolved (SR) and spatial-frequency domain imaging (SFDI). Sun C. et al. (2020) used IS coupled with inverse adding-doubling algorithm for extracting the *μ_*a*_* and *μ_*s*_′* of citrus fruit. Huang et al. (2018) measured the *μ_*a*_* and *μ_*s*_′* of tomatoes at six maturity stages from multichannel hyperspectral imaging-based SR spectra. Vanoli et al. (2020) applied TR and SR spectroscopy to determine the *μ_*a*_* and *μ_*s*_′* of apples after ripening in shelf life. The measured optical properties were used for evaluating the physiochemical properties, such as water content, oil gland size, total soluble solids, titratable acidity, and firmness. All these studies are limited to point measurement, which means the measured optical property is from single point (usually one pixel), and it cannot attain the spatial distribution of optical properties through single measurement. Since plant and food tissues present heterogeneity to some extent, some researchers measured several tissue points and took the average value as the optical property (He et al., 2016). This attempt might partly weaken the effect of measurement location on the intrinsic optical property, but it could not address the issue fundamentally.

Spatial-frequency domain imaging, as an emerging modality for measuring optical properties, is capable of mapping *μ_*a*_* and *μ_*s*_′* on a pixel-by-pixel fashion, which enables to attain 2-D optical property distribution through single measurement. By demodulating the emitted images under structured illuminations with changed frequencies and phases, the *μ_*a*_* and *μ_*s*_′* can be estimated using inverse algorithm based on appropriate light transfer models (Hu et al., 2016). Owing to the capabilities of wide-field and non-contact imaging, and depth-varying and signal-enhanced characterization (Gigan, 2017), SFDI has been applied for measuring the optical property mappings of apple, kiwi, mango, and pear (Hu et al., 2020a; He et al., 2021; Lohner et al., 2021; Sun et al., 2021). However, no reports were found on the prediction of apple quality properties based on the measured optical property mappings. In addition, in the SFDI, one can extract the optical property of single pixel, as well as do the measurements of multiple pixels in a preselected ROI, but the results would present difference to some extent. It is thus necessary to quantify the effect of ROI size (i.e., number of pixels) on optical property measurement. Therefore, the objectives of this research were as (1) to measure the optical property (*μ_*a*_* and *μ_*s*_′*) mappings of apples and compare the optical property difference among different sizes of ROI; (2) to relate the average optical properties to apple SSC, firmness, and color parameters; and (3) to evaluate prediction performance of apple quality properties based on the optical properties using different models.

## Materials and Methods

### Apple Preparation

**FIGURE 1:**
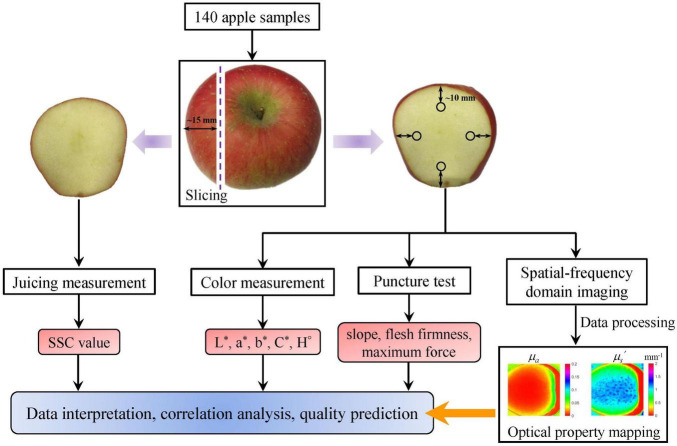
Schematic overview of the experimental and analytical procedures. SSC denotes soluble solid content.

A total of 140 “Golden Delicious” apples with similar size and being free of visible defects were purchased from a local fruit supermarket at the city of Hangzhou. Physical properties (i.e., weight, diameter, and height) of the apples are measured and summarized in Table 1. To complete the acquisition of SFDI and the measurement of quality attributes for all the apples, the experiments were designed and conducted, with the flowchart shown in Figure 1. The intact apples were cut lengthwise to generate a slice with the thickness of about 15 mm. The slice (left in Figure 1) was used for juicing measurement to get the corresponding SSC value, while the other part (right in Figure 1) was subjected to the color measurement, SFDI measurement, and puncture test, respectively, from which the color parameters (L*, a*, b*, C*, and H°), optical property mapping, and firmness parameters [slope, flesh firmness (FF), and maximum force (Max.F)] were obtained. The measured optical property mapping was then used for data interpretation, correlation analysis, and quality prediction. During the experiment, apples were kept at room temperature (∼22°C) with no humidity control and the experiment was completed within 5 days.

**TABLE 1:** Statistics of physical and quality parameters for 140 “Golden Delicious” apples.

Test method	Parameter	Mean	*SD*	Max	Min	CV (%)
Auncel	Weight (g)	226.8	17.9	270.0	190	7.9
Vernier caliper	Diameter (mm)	80.4	2.4	84.9	74.9	2.9
	Height (mm)	68.0	3.7	77.4	59.4	5.4
	Slice thickness (mm)	14.8	0.9	17.2	12.1	5.6
Refractometer	SSC (%)	15.0	1.3	18.6	11.5	8.5
Puncture	Slope (N/mm)	7.3	1.7	11.1	3.4	22.9
	FF (N)	14.0	1.6	17.4	9.5	11.4
	Max.F (N)	16.7	1.2	19.8	13.6	17.5
Colorimeter	L*	76.8	1.1	79.2	74.1	1.4
	a*	−2.9	0.8	−1.1	−5.0	−7.8
	b*	27.3	1.8	34.3	22.6	10.2
	C*	27.4	1.8	34.6	22.8	10.2
	H°	96.1	1.7	100.0	92.4	1.7

*SD, standard deviation; CV, coefficient of variation; FF, flesh firmness; Max.F, maximum force.*

### Acquisition of Spatial-Frequency Domain Imaging and Optical Property Mapping

**FIGURE 2:**
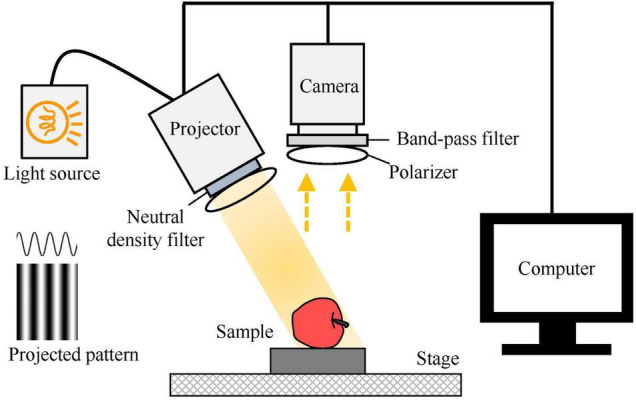
Schematic of a multispectral spatial-frequency domain imaging system for measuring optical property mappings of apples.

Spatial-frequency domain imaging of the apples was acquired using a laboratory-developed SFDI system, as shown in Figure 2. A detailed description of the SFDI system and its calibration procedure were given in our previous study (Hu et al., 2016). The SFDI system mainly consists of a light source, a digital projector, and a color camera. The light source, combined with the DLP-based projector, is used to generate structured patterns for illuminating the apples. The reflected light is received by the camera, which produces SFDI under different frequencies and phases. The neutral density filter mounted with the projector could reduce light intensity uniformly to avoid burning on the apples, while the bandpass filter placed in front of the camera is used for wavelength dispersion, constituting a multispectral SFDI system. A number of two crossed linear polarizers are mounted in the projection and detection arms to reduce specular reflection from the apple surface. In this study, four wavelengths of 460, 527, 630, and 710 nm are used. When spatial frequency of the structured illumination is 0.20 mm^–1^, the light penetration depth within apple tissue is approaching 1 mm (Hu et al., 2021). High-frequency illumination has a relatively shallow light penetration depth, which could be interpreted by the depth-varying feature (Hayakawa et al., 2018; Lu and Lu, 2019). To remove the effect of apple peel (with the thickness about 1 mm) on optical property mapping, six spatial frequencies with the values of 0, 0.04, 0.08, 0.12, 0.16, and 0.20 mm^–1^ were utilized in this study.

**FIGURE 3:**
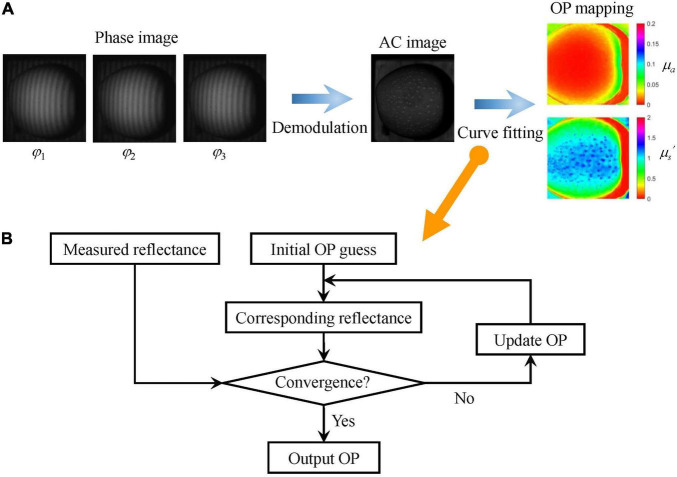
Schematic of optical property mapping using three-phase demodulation coupled with curve fitting **(A)** and flowchart of the curve-fitting algorithm **(B)**. AC and OP denote alternative component and optical property, respectively.

The captured SFDI of apples was processed using appropriate algorithms for extracting optical property mapping, among which image demodulation and inverse estimation are the two key steps (Figure 3A). Great efforts have been made to develop and improve the demodulation and inverse estimation methods (Lu et al., 2016; Hu et al., 2018, 2019; Sun et al., 2022). In this study, the conventional three-phase demodulation coupled with non-linear curve-fitting (TP-CF) method was applied for extracting optical property mapping of apples due to its high accuracy. To implement the TP-CF method, three-phase images at the same frequency were first demodulated to generate one alternative component (AC) image. Then, the non-linear curve-fitting method was used to extract the optical property (*μ_*a*_* and *μ_*s*_′*) mappings of apples on a pixel-by-pixel fashion. It should be noted that two AC images are at least needed for curve fitting. The essential nature of the curve fitting is continuously iterative computing until the difference between the measured reflectance from the AC image and the computed reflectance based on the initially guessed optical properties is within the preset threshold (Figure 3B). In this occasion, dynamic adjustment is carried out to update the initial optical properties until the reflectance present a convergent pattern. To investigate the effect of ROI size on the optical property measurement, four ROIs with different sizes (i.e., 10 × 10, 30 × 30, 60 × 60, and 100 × 100 pixels, corresponding to 3 × 3 mm^2^, 9 × 9 mm^2^, 18 × 18 mm^2^, and 30 × 30 mm^2^ approximately) were selected, and the average value was taken as the optical property.

### Measurement of Apple Quality Attributes

All the 140 apples were subjected to the measurement for different quality attributes. Juice of apple fruit was extracted from the apple slice (left in Figure 1), and SSC was measured using a handheld digital refractometer (model PR-101, Atago Co., Tokyo, Japan). Apple color was measured from the other part (right in Figure 1) using a colorimeter (CR-400, Konica Minolta Sensing, Inc., Tokyo, Japan) for the L*, a*, b*, C*, and H° values in triplicate, and average values were recorded for further analysis. As shown in Figure 1, apple firmness was measured from the surface area in quadruplicate with the distance between the center of puncture points and the external contour of apple about 10 mm, and the average values were recorded. Puncture tests were carried out using the texture analyzer (Model TA.XT plus, Stable Micro System, United Kingdom) equipped with a 5-mm cylindrical Magness-Taylor probe at a test speed of 4 mm/s and the penetration depth was set as 8 mm. A number of three parameters related to the firmness, which include slope in N/mm, FF in N, and Max.F in N, were then determined from the force–displacement curve. The slope was measured between the point of initial displacement and the point corresponding to the Max.F, while the FF was defined as the average force over the puncture distances between the rupture point and maximum puncture depth (8 mm).

### Correlation Analysis and Prediction Modeling

Pearson’s linear correlation analysis was performed on the relationship among all quality parameters and the absorption and reduced scattering coefficients as well as their combinations (i.e., *μ_*a*_*, *μ_*s*_′*, *μ_*a*_* × *μ_*s*_′*, and *μ_*eff*_* = [3*μ_*a*_* (*μ_*a*_* + *μ_*s*_′*)]^1/2^) using the software of IBM SPSS Statistics 25. *μ_*a*_* × *μ_*s*_′* is the multiplication of *μ_*a*_* and *μ_*s*_′*, and it was reported to be more effective for predicting quality of tomatoes than using individual *μ_*a*_* and *μ_*s*_′* (Huang et al., 2018). *μ_*eff*_* is a useful parameter for comparing light transmittance characteristics of different tissues and their wavelength dependence (Flock et al., 1987). To further explore the relationship between optical properties and quality parameters of apples (i.e., SSC, firmness and color), prediction models were established based on *μ_*a*_*, *μ_*s*_′*, *μ_*a*_* × *μ_*s*_′*, and *μ_*eff*_* using support vector machine (SVM), multiple linear regression (MLR), and partial least squares (PLS) at the four wavelengths. The models of SVM, MLR, and PLS were developed by the Unscrambler X 10.4 software. All the 140 apple samples were randomly divided with 75% for the calibration set and the remaining 25% for the validation set. The performance of the prediction models was evaluated using the statistic parameters of correlation coefficient of prediction (r_p_) and root mean square error of prediction (RMSEP).

## Results and Discussion

### Statistics of Measured Quality Parameters

The statistical data of SSC, firmness (slope, FF, Max.F), and color (L*, a*, b*, C*, and H°) for all tested apples are summarized in Table 1. There was no relatively large variation for all the nine quality indexes based on the standard deviation (SD) and coefficient of variation (CV), since all the 140 apples were purchased from the same batch with similar properties. It was observed that the distributions of apple SSC and color were relatively narrow than the firmness, with the CV values close to or smaller than 10%. This observation brought great challenges for predicting SSC and color parameters, which will be discussed in the following section.

Correlations among the SSC, firmness, and color parameters for all apples are summarized in Table 2. It was observed that the overall correlation among these quality parameters was not strong, with most of the coefficients smaller than 0.50. This might be due to the fact that the apples used in this study were similar in appearance and had no difference in maturation stage or shelf life, thus causing narrow distribution of quality attributes. It should be noted that the correlation coefficient between the Max.F and FF was as high as 0.80, because the FF was defined as the average force over the puncture distances between the rupture point (Max.F) and puncture depth. This phenomenon was also reported by Huang et al. (2018) when studying tomato fruit based on spatially resolved spectroscopy. The correlation coefficient between b* and C* was 1.00, because the two parameters were very close to each other.

**TABLE 2:** Pearson’s linear correlation coefficients among apple quality parameters.

Parameter	SSC	Slope	FF	Max.F	L*	a*	b*	C*	H°
SSC	1.00								
Slope	0.46	1.00							
FF	**0.51**	0.38	1.00						
Max.F	**0.69**	**0.51**	**0.80**	1.00					
L*	0.32	0.36	0.36	0.36	1.00				
a*	**0.77**	−0.45	−0.32	0.31	−0.48	1.00			
b*	**0.51**	0.48	0.42	**0.53**	−**0.50**	−**0.50**	1.00		
C*	0.49	0.48	0.42	**0.52**	−0.49	−**0.52**	**1.00**	1.00	
H°	−**0.86**	0.38	−0.34	−0.40	**0.57**	−**0.91**	−0.49	−0.46	1.00

*Significant correlations are in bold (n = 140, r ≥ 0.5, p-value ≤ 0.05).*

*SSC, soluble solid content; FF, flesh firmness; Max.F, maximum force.*

### Optical Property Mappings of Apples

**FIGURE 4:**
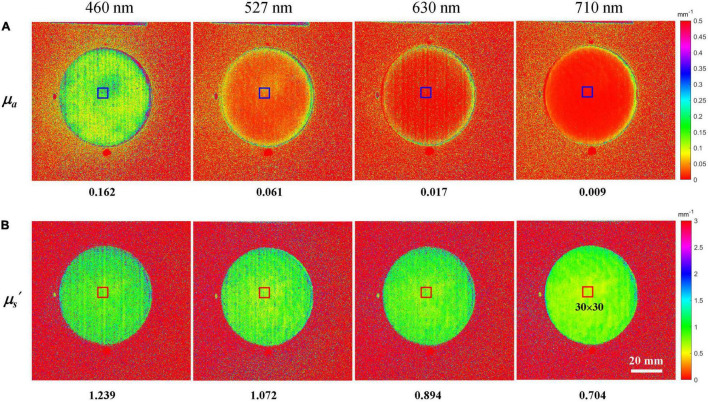
Absorption coefficient **(A)** and reduced scattering coefficient **(B)** mappings of a representative apple at the wavelengths of 460, 527, 630, and 710 nm.

To determine the optical property mappings of apples, the TP-CF method was carried out, and the resulting absorption and scattering properties are displayed in Figures 4A,B, respectively. The numbers labeled below the sub-pictures are the average values of *μ_*a*_* or *μ_*s*_′* for the marked ROI with the size of 30 × 30 pixels. It should be noted that the circular part is sliced apple tissue with relatively flat surface, while the surrounding part, which has a more uniform color distribution, is the sample holder made of aluminum material with blackening treatment. There was a decreased tendency of the *μ_*a*_* and *μ_*s*_′* along the four wavelengths. Comparing the scattering properties at a wavelength, like 527 nm in Figure 4B, a spatial variation of *μ_*s*_′* for the apple tissue between 0.996 and 1.152 mm^–1^ was noticed. The complex apple tissue formed by different components with different physicochemical characteristics and the performance of structured illumination (e.g., non-uniformity), as well as data processing algorithm for optical property estimation, are the potential factors that cause the variation. A similar variation also occurs for the *μ_*a*_* mappings in Figure 4A. It was observed that the spatial variation of optical properties at 710 nm was less pronounced than the other three wavelengths, which demonstrates that wavelength plays a non-negligible role when estimating apple optical properties.

**FIGURE 5:**
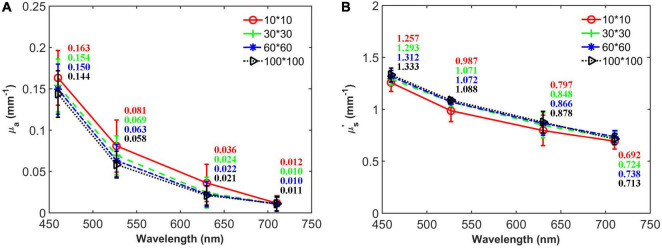
Average absorption coefficient **(A)** and reduced scattering coefficient **(B)** of a representative apple with different sizes of ROI at the wavelengths of 460, 527, 630, and 710 nm.

Figure 5 shows the average optical properties of four different ROI with the sizes of 10 × 10, 30 × 30, 60 × 60, and 100 × 100 along the four wavelengths. The *μ_*a*_* and *μ_*s*_′* for different sizes of ROI are quite similar, but still, a difference can be observed. In general, the optical property value in small size ROI (10 × 10) was slightly different from the value in large size ROI, which might be caused by the spatial variation mentioned above. For the three larger ROIs, the average optical properties were close to each other, with the differences of 6.94–19.0% and 1.56–3.51% for *μ_*a*_* and *μ_*s*_′*, respectively. Considering the fact that larger-size ROI means more cost of time, the ROI with the size of 30 × 30 was chosen for averaging the *μ_*a*_* and *μ_*s*_′* and would be used in the following sections.

To our knowledge, this is the first time that the optical properties of apple tissue were determined from different sizes of ROI. Based on SFDI measurements of “Braeburn” apples, Lohner et al. (2021) recently observed that *μ_*a*_* was about 0.01–0.04 mm^–1^ for the cortex tissue at 656 nm, which covers the *μ_*a*_* range at 630 nm in this study (0.021–0.024 mm^–1^), if the value from the ROI of 10 × 10 is excluded. As known from the previous work, the wavelength of 656 nm is mainly related to the presence of chlorophyll *b* (Merzlyak and Solovchenko, 2002), and it is thus reasonable that the *μ_*a*_* value is larger than the present results. Our results are also comparable to the published integrating sphere measurements by other researchers. For example, Wei et al. (2020) measured the optical properties of cold-stored “Fuji” apples over 400–1,100 nm and reported that the *μ_*a*_* was in the range of about 0.02–0.13 mm^–1^, whereas the *μ_*s*_′* was between 1.0 and 1.75 mm^–1^ for the flesh tissue, which are comparable with the present results.

### Correlations Between Optical Properties and Apple Quality Attributes

Table 3 presents the relationships between the average optical properties and apple quality parameters. The correlations between the optical properties and their combinations at 527 and 630 nm measured by SFDI and apple quality parameters are not reported as non-significant. Better correlations were observed between *μ_*a*_* and quality parameters, compared with *μ_*s*_′*, which is in general agreement with the previous studies for apples, tomatoes, and peaches (Do Trong et al., 2014; Huang et al., 2018; Sun Y. et al., 2020). The *μ_*a*_*__460_ was negatively related to a* and C* and positively related to b*, slope, FF, and Max.F, whereas *μ_*a*_*__710_ was only positively related to b* and negatively related to a*, C*, slope, FF, and Max.F. As for scattering, no significant correlations were found between *μ_*s*_′*__460_ and quality parameters, whereas *μ_*s*_′*__710_ was positively and negatively related to b* and C*, respectively. It was observed that the combinations of *μ_*a*_* × *μ_*s*_′*__460_, *μ_*a*_* × *μ_*s*_′*__710_, *μ_*eff*_*__460_, and *μ_*eff*_*__710_ resulted in better correlation results, compared with the individual optical properties. This is especially true for the correlations between the color parameters of a*, b*, C*, and *μ_*a*_* × *μ_*s*_′*__460_, *μ_*a*_* × *μ_*s*_′*__710_. For example, using *μ_*a*_* × *μ_*s*_′*__710_ improved correlation coefficients of a*, b*, and C* by 14.8, 25.4, and 25.4%, compared with the correlation analysis based on the *μ_*a*_*__710_. Moreover, a noticeable improvement was found for SSC when using the combinations of optical properties (Table 3). These results demonstrated that optical property measurement can provide useful information about the apple quality.

**TABLE 3:** Pearson’s linear correlation coefficients between optical properties and apple quality parameters.

	*μ_*a*_*__460_	*μ_*a*_*__710_	*μ_*s*_′*__460_	*μ_*s*_′*__710_	*μ_*a*_* × *μ_*s*_′*__460_	*μ_*a*_* × *μ_*s*_′*__710_	*μ_*eff*_*__460_	*μ_*eff*_*__710_
SSC	–0.42	–0.45	–0.31	–0.39	−**0.61**	−**0.65**	−**0.61**	−**0.64**
Slope	**0.64**	−**0.55**	0.40	0.45	**0.63**	−**0.52**	**0.64**	−**0.53**
FF	**0.61**	−**0.56**	0.33	0.34	**0.69**	−**0.65**	**0.69**	−**0.65**
Max.F	**0.62**	−**0.59**	0.40	0.38	**0.62**	−**0.60**	**0.63**	−**0.59**
L*	0.39	0.32	0.21	0.32	0.49	0.37	0.30	0.28
a*	−**0.57**	−**0.54**	–0.23	–0.20	−**0.65**	−**0.62**	−**0.57**	−**0.61**
b*	**0.59**	**0.59**	0.32	**0.52**	**0.67**	**0.74**	**0.60**	**0.67**
C*	−**0.59**	−**0.59**	–0.32	−**0.52**	−**0.67**	−**0.74**	−**0.60**	−**0.66**
H°	0.36	0.26	0.15	0.11	0.45	**0.50**	0.26	0.28

*Significant correlations are in bold (n = 140, r ≥ 0.5, p-value ≤ 0.05).*

*SSC, soluble solid content; FF, flesh firmness; Max.F, maximum force.*

### Prediction for Apple Quality Attributes

The resulting r_p_ and RMSEP from SVM, MLR, and PLS based on *μ_*a*_*, *μ_*s*_′*, *μ_*a*_* × *μ_*s*_′*, and *μ_*eff*_* are summarized in Table 4. Overall, the prediction result performs not well with the *r*_p_-values of 0.24–0.86 and 0.19–0.82 for the SVM and MLR, respectively. The result predicted by PLS is worse and not reported. It could be observed that color parameters of b* and C* achieved the best prediction performance (*r*_p_ = 0.86), followed by the firmness parameters with the *r*_p_-values of 0.68, 0.73, and 0.79 for slope, FF, and Max.F, and finally, SSC was the most challenging for the prediction (*r*_p_ = 0.66). The prediction results for all the apple quality parameters based on *μ_*a*_* were far better than that based on *μ_*s*_′*, which is consistent with the findings of Pearson’s correlation analysis above. The combinations of *μ_*a*_* × *μ_*s*_′* and *μ_*eff*_* gave better prediction results, compared to the individual *μ_*a*_* or *μ_*s*_′*. For example, using *μ_*a*_* × *μ_*s*_′* for predicting the parameter of slope by the SVM resulted in *r*_p_ of 0.68, which represents 17.2% improvement, compared with the individual *μ_*a*_*. It should be mentioned that the combinations improved the prediction of SSC significantly (> 34.0%), which also agrees well with the correlation analysis.

**TABLE 4:** SVM and MLR prediction results for apple quality parameters based on absorption coefficient (*μ_*a*_*), reduced scattering coefficient (*μ_*s*_′*), and their combinations.

Quality parameter	Statistic parameter	SVM prediction				MLR prediction			
		*μ_*a*_*	*μ_*s*_′*	*μ_*a*_* × *μ_*s*_′*	*μ_*eff*_*	*μ_*a*_*	*μ_*s*_′*	*μ_*a*_* × *μ_*s*_′*	*μ_*eff*_*
SSC	r_p_	0.47	0.30	0.66	0.63	0.40	0.31	0.59	0.60
	RMSEP	1.28	1.27	1.28	1.28	1.32	1.34	1.32	1.32
Slope	r_p_	0.58	0.32	0.68	0.67	0.54	0.27	0.60	0.61
	RMSEP	1.65	1.63	1.66	1.66	1.70	1.72	1.71	1.71
FF	r_p_	0.64	0.24	0.73	0.72	0.53	0.20	0.63	0.62
	RMSEP	2.24	2.29	2.24	2.25	2.25	2.29	2.25	2.25
Max.F	r_p_	0.72	0.25	0.79	0.78	0.56	0.21	0.64	0.64
	RMSEP	1.20	1.27	1.21	1.22	1.28	1.31	1.29	1.29
L*	r_p_	0.34	0.35	0.41	0.42	0.31	0.33	0.38	0.40
	RMSEP	1.06	1.01	1.07	1.07	1.09	1.09	1.10	1.10
a*	r_p_	0.57	0.35	0.66	0.57	0.52	0.28	0.58	0.50
	RMSEP	0.84	0.82	0.84	0.84	0.86	0.85	0.87	0.87
b*	r_p_	0.80	0.61	0.85	0.86	0.75	0.57	0.78	0.82
	RMSEP	2.32	2.42	2.16	2.28	2.52	2.53	2.35	2.44
C*	r_p_	0.81	0.61	0.85	0.86	0.75	0.58	0.77	0.82
	RMSEP	2.32	2.42	2.16	2.28	2.53	2.53	2.35	2.44
H°	r_p_	0.33	0.26	0.38	0.37	0.31	0.19	0.33	0.32
	RMSEP	1.73	1.71	1.70	1.71	1.76	1.78	1.75	1.76

*SSC, soluble solid content; FF, flesh firmness; Max.F, maximum force; r_p_, correlation coefficient of prediction; RMSEP, root mean square error of prediction.*

## Discussion

Optical property measurement, as a powerful tool for interpreting light-tissue interaction, as well as an alternative solution for quality assessment of plant and food products by quantitatively separating absorption and scattering information from spectroscopic and/or image signals, has received increasing interests. Many fruits and vegetables, such as apple, pear, peach, citrus fruit, tomato, onion, and sweet potato, have been tested using diverse optical measurement techniques (Hu et al., 2015, 2020b; Lu et al., 2020). However, most of the efforts were made to obtain the optical property of single point (usually one pixel in the tissue). To our knowledge, SFDI is the sole technique which is capable of measuring the optical property mappings (in the axial and transverse directions), and till now, only apple and pear fruit have been studied (Hu et al., 2020a; He et al., 2021; Lohner et al., 2021). The apple flesh, as a relatively uniform tissue, should present homogeneous optical property mapping, which was confirmed by the results observed in this paper. For non-uniform samples, such as apple with peel, optical property mapping is critical to elaborate the spatial distribution of optical properties, which, in turn, would help to select the optimal location in optical inspection for quality evaluation.

It is well known that there are several peaks in apple absorbance spectra, which can be attributed to carotenoids around 500 nm, chlorophyll around 670 nm, and water around 980 nm (Vanoli et al., 2020). The wavelengths of 460 and 710 nm, under which better correlations between the optical properties and apple quality parameters were obtained, are close to the absorption peaks of carotenoids and chlorophyll, respectively. However, apple preparation in this study is destructive by removing a slice of about 15 mm, and the color parameters are all measured from the flesh tissues. Though pigments could contribute to flesh coloration, the amounts of carotenoids and chlorophylls in the flesh are very low (Delgado-Pelayo et al., 2014), which could partly explain why the correlations between optical property and color parameters were not high enough.

This study showed that absorption properties provided useful information for apple SSC, firmness, and color prediction, while the prediction performance based on the scattering properties was much worse. This observation is general agreement with the previous studies for apple, peach, and tomato using the spatially resolved technique (Do Trong et al., 2014; Huang et al., 2018; Sun Y. et al., 2020). For SSC and color parameters, they are closely related to the chemical compositions and pigment contents in apples, which have direct relationships with the absorption properties. On the other hand, and surprisingly enough, the prediction of firmness parameters based on the absorption properties was far better than that based on the scattering properties. This unexpected outcome could be attributed to the fact that SFDI is limited to light penetration depth (2–3 mm) (Lu and Lu, 2019; Hu et al., 2021), while the firmness measurement by the texture analyzer probed the flesh much deeper (8 mm). In addition, the wavelengths used in this study are near to the characteristic wavelengths with absorption peaks, while the scattering properties have no features in those wavelengths. This may also explain why absorption properties performed better correlations with apple quality parameters than scattering properties. Since the apple quality is accompanied with both chemical compositions and tissue microstructures, the combinations of optical properties could improve the prediction performance.

The prediction models based on SVM were superior to the models established by MLR and PLS. This may be attributed to the fact that four wavelengths are not adequate to establish the stable and adaptable MLR and PLS models, while SVM is one of machine-learning methods, which is more powerful in dealing with this issue. This observation suggests that other machine-learning and deep learning methods, such as artificial neural network and convolutional neural network, can be tested for quality prediction based on optical properties in the future. Overall, the correlation and prediction performance between the optical properties and quality parameters were comparable to the results of some published work. For instance, He et al. (2016) and Sun C. et al. (2020) showed that the prediction models for SSC of pear and citrus fruit based on the *μ_*a*_* spectra measured by integrating sphere technique gave the *r*_p_-values of 0.63 and 0.67, respectively, while the r_p_ for SSC of tomato fruit based on the absorption properties estimated from spatially resolved diffuse reflectance was 0.62 (Huang et al., 2018), which are all comparable to the *r*_p_ of 0.66 in this study. As for the firmness prediction, He et al. (2016) reported that the PLS model gave *r*_p_-value of 0.66, which was less satisfied than the results of Do Trong et al. (2014) (*r*_p_ = 0.84). The apple color prediction in this study was much better than the result reported by Vanoli et al. (2020) based on time- and spatially resolved techniques. Though the prediction performance of these results showed great differences, which may be in part caused by the factors of cultivar, growing, maturity and storage conditions, and measuring technique, they all demonstrated that there was a great potential for quality prediction based on the measured optical properties. However, in this paper, the results are still far away from practical application in the field of non-destructive inspection. Very few samples and characteristic wavelengths, as well as the narrow distribution of quality attributes (SSC, firmness, and color) for the apple samples used in this study, are the critical factors that result in low *r*_p_-values. Furthermore, relatively low correlation for SSC could also be caused by the different locations (i.e., the left and right pieces in Figure 1) for juicing and SFDI measurements. Apples were cut into two parts in the sample preparation to generate a relatively smooth surface, which is critical to keep the distance between camera and sample surface consistent during SFDI measurement. However, this operation could not meet the requirement of non-destructive inspection in practical applications. Apple contour has non-negligible effect on optical property mapping, and thus, the contour correction is worth studying in the future research.

## Conclusion

The optical property mappings of “Golden Delicious” apples were measured using the SFDI technique at the wavelengths of 460, 527, 630, and 710 nm. Spatial variations in the absorption coefficient (*μ_*a*_*) and reduced scattering coefficient (*μ_*s*_′*) mappings for the sliced flesh tissue were noticed, but overall, the *μ_*a*_* and *μ_*s*_′* distributions were relatively uniform. Different sizes of ROI (10 × 10, 30 × 30, 60 × 60, and 100 × 100 pixels) had less effect on the average *μ_*a*_* and *μ_*s*_′*, except for the 10 × 10 that was too small. The average *μ_*a*_* and *μ_*s*_′* showed a decreased tendency along the four wavelengths. Correlations between apple quality parameters and *μ_*a*_* and *μ_*s*_′*, as well as their combinations (*μ_*a*_* × *μ_*s*_′* and *μ_*eff*_*) at 460 and 710 nm, were superior to those at 527 and 630 nm, since the former two wavelengths were close to the absorption peaks. The prediction for quality parameters (SSC, firmness, and color) based on the *μ_*a*_* was far better than that based on the *μ_*s*_′*, and the combinations of *μ_*a*_* × *μ_*s*_′* and *μ_*eff*_* improved the prediction performance, compared to the individual *μ_*a*_* or *μ_*s*_′*. The prediction models established based on SVM outperformed those by MLR and PLS. The best prediction models for SSC, slope, FF, and Max.F were all achieved based on the *μ_*a*_* × *μ_*s*_′*, with the correlation coefficients of prediction (*r*_p_) of 0.66, 0.68, 0.73, and 0.79, respectively, whereas the *μ_*eff*_* gave the best prediction for most of the color parameters, with the *r*_p_-value of 0.86 for both of the parameters of b* and C*.

## Data Availability Statement

The original contributions presented in the study are included in the article/supplementary material, further inquiries can be directed to the corresponding author/s.

## Author Contributions

HP: conceptualization, data analysis, writing, reviewing, editing, and project administration. CZ: data collection, methodology, reviewing, and editing. ZS: data analysis, reviewing, and editing. TS and JW: reviewing and editing. DH: conceptualization, methodology, data collection, final analysis, writing—original draft preparation, reviewing, and editing. ZY: resources, reviewing, and editing. All authors contributed to the article and approved the submitted version.

## Conflict of Interest

The authors declare that the research was conducted in the absence of any commercial or financial relationships that could be construed as a potential conflict of interest.

## Publisher’s Note

All claims expressed in this article are solely those of the authors and do not necessarily represent those of their affiliated organizations, or those of the publisher, the editors and the reviewers. Any product that may be evaluated in this article, or claim that may be made by its manufacturer, is not guaranteed or endorsed by the publisher.
